# Fertility after photodynamic inactivation of bacteria in extended boar semen

**DOI:** 10.3389/fmicb.2024.1429749

**Published:** 2024-08-07

**Authors:** Anne-Marie Luther, Mohammad Varzandeh, Christina Beckermann, Leon Feyer, Isabel Katharina Maaßen, Harriёtte Oldenhof, Steffen Hackbarth, Dagmar Waberski

**Affiliations:** ^1^Unit for Reproductive Medicine/Clinic for Swine and Small Ruminants, University of Veterinary Medicine Hannover, Hannover, Germany; ^2^Photobiophysics, Institute of Physics, Humboldt University of Berlin, Berlin, Germany; ^3^Unit for Reproductive Medicine/Clinic for Horses, University of Veterinary Medicine Hannover, Hannover, Germany

**Keywords:** photodynamic inactivation, photosensitizer, singlet oxygen, sperm, boar, bacteria, semen preservation

## Abstract

Antimicrobial resistance is an increasing challenge in semen preservation of breeding animals, especially in the porcine species. Bacteria are a natural component of semen, and their growth should be inhibited to protect sperm fertilizing capacity and the female’s health. In pig breeding, where semen is routinely stored at 17°C in the liquid state, alternatives to conventional antibiotics are urgently needed. Photodynamic inactivation (PDI) of bacteria is a well-established tool in medicine and the food industry but this technology has not been widely adopted in semen preservation. The specific challenge in this setting is to selectively inactivate bacteria while maintaining sperm integrity and functionality. The aim of this study was to test the principle of PDI in liquid stored boar semen using the photosensitizer 5,10,15,20-tetrakis(N-methyl-4-pyridyl)-21H,23H-porphine (TMPyP) and a white light LED-setup. In the first step, photophysical experiments comprising singlet oxygen phosphorescence kinetics of TMPyP and determination of the photosensitizer triplet time revealed a sufficiently high production of reactive singlet oxygen in the Androstar Premium semen extender, whereas seminal plasma acted as strong quencher. *In vitro* experiments with extended boar semen showed that the established PDI protocol preserves sperm motility, membrane integrity, DNA integrity, and mitochondrial activity while efficiently reducing the bacteria below the detection limit. A proof-of-concept insemination study confirmed the *in vivo* fertility of semen after photodynamic treatment. In conclusion, using the PDI approach, an innovative tool was established that efficiently controls bacteria growth in extended boar and maintains sperm fertility. This could be a promising contribution to the One Health concept with the potential to reduce antimicrobial resistance in animal husbandry.

## 1 Introduction

Artificial insemination (AI) is a widely used biotechnology in pig reproduction. Traditionally, semen is stored in a liquid state up to 7 days between 16 and 18°C. The relatively high temperature is regarded as the optimum for boar sperm survival but poses a risk for bacterial growth. Bacteria, mostly gram-negative opportunistic pathogens belonging to *Enterobacteriaceae* (Althouse and Lu, 2005), are an inevitable component of boar semen and have thus contributed to the overuse of antibiotics in semen extenders (Schulze et al., 2020).The loss of antimicrobial efficiency and national bans on still effective antibiotics in semen extenders promote the search for alternative antimicrobial concepts in boar semen preservation (Waberski et al., 2019; Schulze et al., 2020). Besides having broad-spectrum antimicrobial activity, alternative concepts must fulfill several requirements: they should not be harmful to the sperm, sow, or the environment and should be easily applicable in aqueous media. Moreover, there should not be a risk for the development of resistance. To date, only the recently established cold-storage of boar semen at 5°C fulfills these requirements and has shown effectiveness in field insemination trials (Waberski and Luther, 2024). However, reservations about changing traditional temperature management are driving research into alternative antimicrobial concepts of semen storage at 17°C. Among these, extender additives such as antimicrobial proteins, biocompounds, plant extracts, and nanoparticles have been tested as well as mechanical-physical decontamination techniques, including single layer centrifugation (SLC), and microfiltration [reviewed by Ďuračka et al. (2023)], with recorded field usage being reported for SLC (Ngo et al., 2023). Until now, none of the aforementioned tools has been implemented into insemination practices, mostly due to insufficient antibacterial efficiency, sperm damage, limited practical use, or high costs. Hence, the search for further alternative concepts to conventional antibiotics in boar semen preservation remains important.

**Figure 1:**
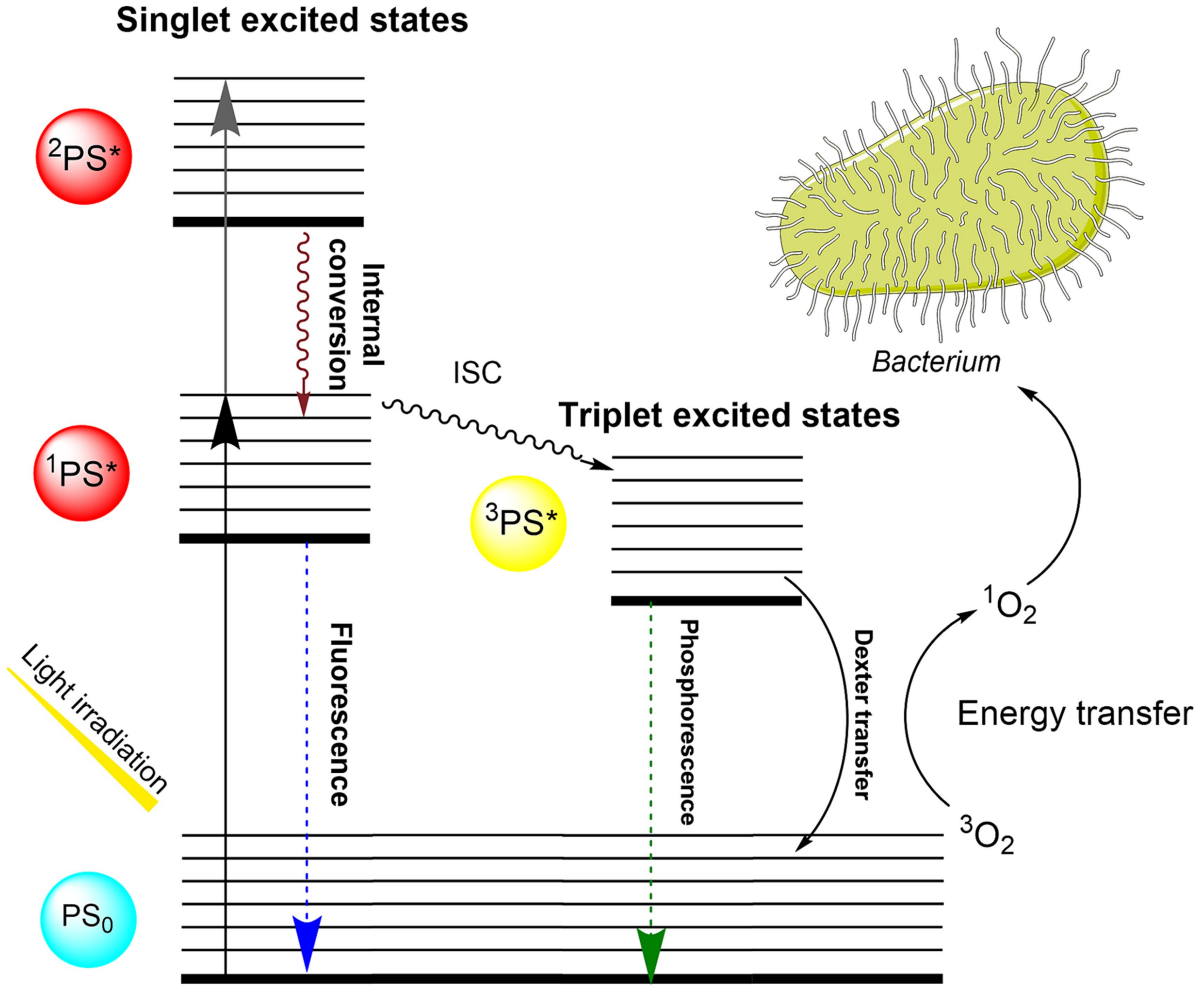
Jablonski diagram describing the photophysical and photochemical mechanisms leading to the generation of singlet oxygen against bacteria. Upon absorbing light, the TMPyP photosensitizer is excited from its ground singlet state (PS_0_) to excited singlet states (^1^PS*, ^2^PS*). The excited photosensitizer can decay to the S_0_ by fluorescence light emission or can undergo intersystem crossing (ISC) to convert into the long-lived triplet state (^3^PS*) (Castano et al., 2005). Photosensitizer in triplet state can engage in a Type II reaction, wherein it transfers energy to molecular oxygen through Dexter transfer, resulting in the generation of singlet oxygen (^1^O_2_). Singlet oxygen acts as an oxidizing agent against the bacterial membrane, leading to the inactivation (Muehler et al., 2022).

Photodynamic inactivation (PDI) is an effective tool against gram-positive and gram-negative bacteria, viruses, and fungi and has the potential to replace antibiotics in many areas (Pohl et al., 2016). The principle of action relies on the combined effect of a photosensitizer (PS), molecular oxygen, and light. By exposure to visible light, the absorbed light energy is transferred to adjacent molecular oxygen and results in the generating of singlet oxygen. This highly reactive oxygen species (ROS) attacks bacterial cell wall components and hence causes instant, irreversible oxidative damage to the microorganisms (Eckl et al., 2018). Details of the photophysical and photochemical mechanisms leading to the generation of singlet oxygen against bacteria are shown in the Jablonski diagram (Figure 1).

The photodynamic treatment has developed as an alternative therapy of skin lesions (de Oliveira et al., 2022) and cancer (Jiang et al., 2023) in human medicine and has also found use for decontamination in the food industry (Ghate et al., 2019) and in wastewater management (Ndlovu et al., 2023). Attempts to make use of this technology for the decontamination of animal semen were less successful in bovine (Eaglesome et al., 1994) and avian species (Novaes et al., 2023) due to incomplete reduction of bacteria and/or an increase in sperm damage. In contrast, Oliveira et al. (2020) reported the elimination of bovine alphaherpesvirus 1 (BoHV-1) using 10 μM of the PS zinc-tetracarboxy-phthalocyanine and hematoporphyrin conjugated to Immunoglobulin Y (IgY) anti-BoHV-1 in cell cultures without affecting the sperm motility and morphology in fresh bull semen. In the context of semen preservation, the specific challenge of PDI is to selectively kill bacteria without harming the sperm. For the application of PDI in semen, it is essential to ensure no toxicity from the PS (in the absence of light) or from light (in the absence of PS) to the spermatozoa. A frequently used and promising candidate is the cationic PS 5,10,15,20-tetrakis(1-methylpyridinium-4-yl) porphyrin (TMPyP). The PS TMPyP has proven to be ineffective against mammalian cells while impressively inhibiting the growth of gram-negative and gram-positive bacteria and is commercially available at a low cost and does not harm the environment (Pohl et al., 2016). In addition to the choice and concentration of PS and the light setup, the potential presence of quenching molecules in seminal plasma (SP) and/or extender components needs to be considered for the efficacy of the PDI in animal semen.

Taking all these challenges into account, the aim of the present study was to establish and test a PDI technique for use in extended boar semen. This is an innovative approach in an attempt to counteract emerging antimicrobial resistance in animal semen. To achieve this, a series of photophysical, microbiological, and spermatological experiments were conducted, followed by an *in vivo* fertility test to prove the concept.

## 2 Materials and methods

### 2.1 Photosensitizer and illumination setup

**Figure 2:**
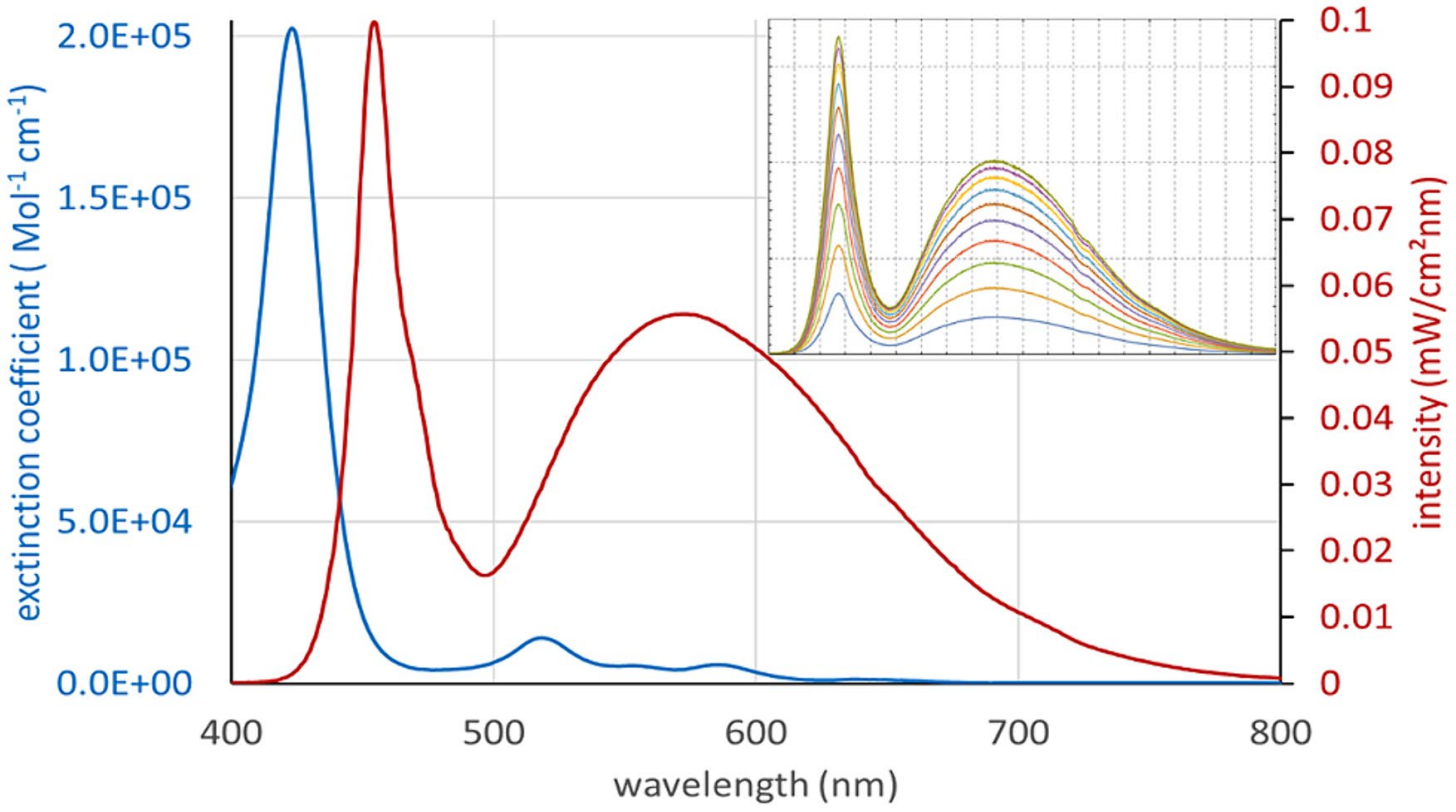
Spectrum of the white light illumination setup (red), which overlaps well with the extinction spectrum of TMPyP except the Soret band. The insert shows the spectra at different current settings for the white light LEDs.

**Figure 3:**
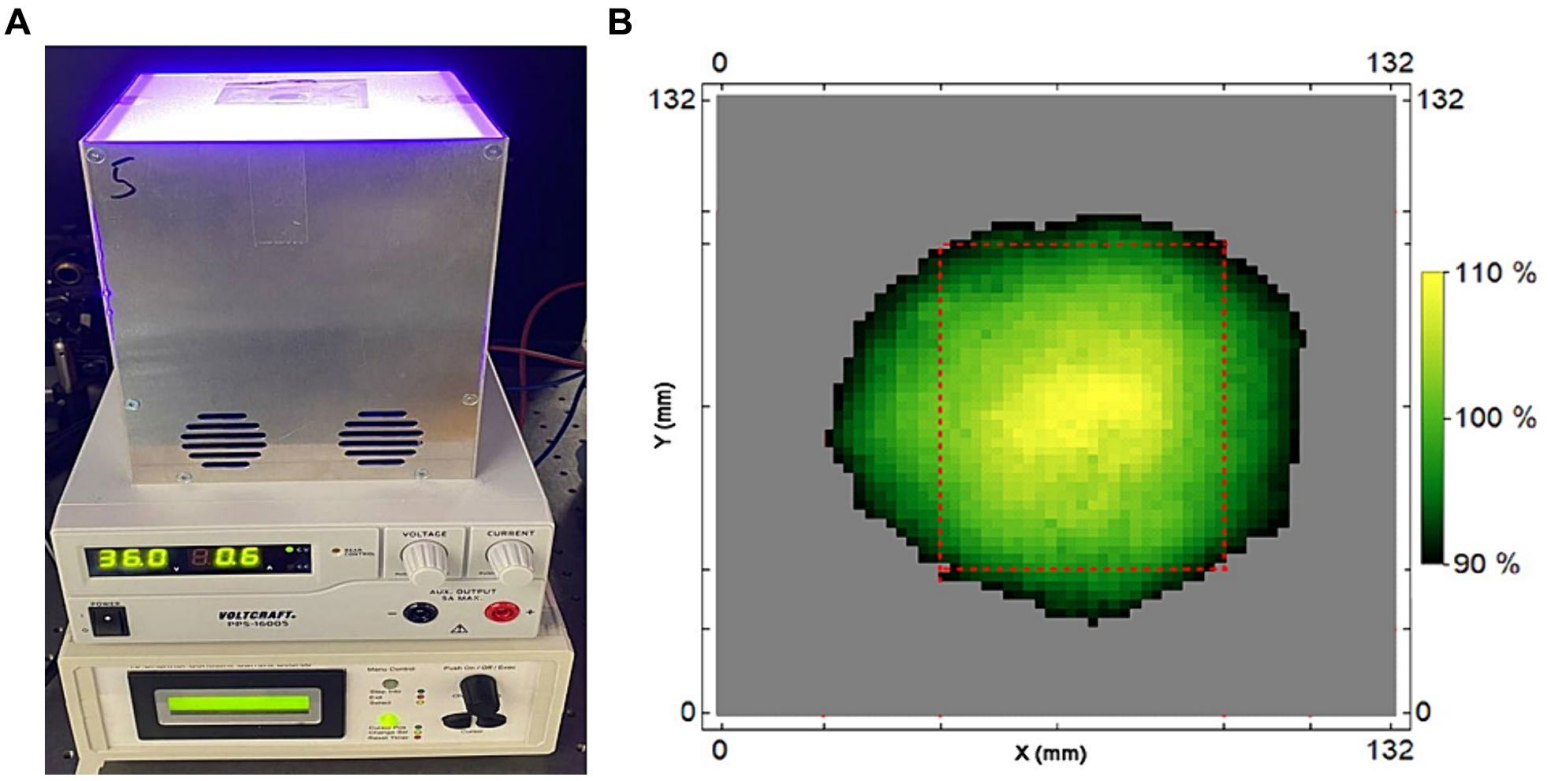
**(A)** Setup for defined illumination of the samples under investigation (top to bottom: Lamp with sample bag, power supply, 10 channel current controller). Correctly placed, the full sample volume lies within the illumination surface for which the intensity deviates less than 10% from the indicated intensity. **(B)**: Heatmap with a 2 × 2 mm^2^ resolution for the local intensity of the lamp, here shown for 500 mA current setting. The outer dimensions of the sample bags are indicated by red dashed lines.

The photosensitizer (PS) TMPyP was used in extended semen filled into transparent plastics bags (Quick Tip narrow type 90 mL, Minitüb GmbH, Tiefenbach, Germany) prepared with dimensions 5.2 × 7 cm. The sample size was 8 mL, resulting in a thickness of 5 mm in the central part of the sample bag. Defined illumination was achieved using LED white light lamps with broad spectral emission, which can be dimmed by controlling the electrical current without large changes in the spectrum. The spectrum of the white light illumination setup overlaps well with the extinction spectrum of TMPyP (Figure 2). However, the Soret band of the PS is barely covered by the lamp. Thus, there are low intensity gradients and spectrum changes during illumination across the sample. The setup for defined illumination of the semen sample is shown in Figure 3A. The intensity heatmap (Figure 3B) indicates a deviation of less than 10% from the indicated intensity at all locations of the sample bag. Using TMPyP with a concentration of 2 μM, the intensity reduction across the sample bag by TMPyP was below 3% for all wavelengths down to 450 nm. All in all, the efficient illumination intensity is described as (0.83 ± 0.10) * I_set_, where I_Set_ denotes the corresponding Intensity per cm^2^ at the surface of the illumination area. Table 1 shows the Current settings (I) for the LED driver and the corresponding values of I_set_.

**Table 1:** Current setting (I) for the LED driver and the corresponding values of I_Set_.

I in mA	50	100	200	300	400	500	600	700	800	900	1,000
I_Set_ in mW/cm^2^	0.98	1.83	3.3	4.5	5.6	6.6	7.4	8.1	8.7	9.2	9.5

Since both the absorption of the dye and the intensity of the illumination change with the wavelength, intensities for white light illumination are difficult to compare.

Therefore, the photon-based description of the absorbed light was used to enable comparison with other light sources, like lasers (Equation 1).


(1) $$n_{\mathrm{abs}}=\frac{1}{hc}\overset{\lambda_{1}}{\underset{\lambda_{2}}{\int }}I\left(\lambda \right).\left(1-10^{-A\left(\lambda \right)}\right).\lambda d\lambda$$

*I*(*λ*) is the intensity per cm^2^ and *A*(*λ*) the absorbance of the TMPyP. Strictly speaking, this formula does not account for scattering, but since highly diluted samples were used in this study, the differences are smaller than the error margin we previously described.

For a 2 μM concentration of TMPyP in a 10% dilution of seminal plasma in BTS, identical photosensitization will be obtained when the sample is illuminated with 1.3 mW/cm^2^ at 405 nm or 8.5 mW/cm^2^ at 532 nm.

### 2.2 Exclusion of background light effects

It was impressively demonstrated that TMPyP is a powerful PS that can promote phototoxic effects even under low background light, thus posing a risk for falsifying results (Eckl et al., 2018). Our experiments required some background room light to ensure proper sample handling. To exclude the effect of (white) background light, we designed a special dark room lamp emitting red light. It consisted of a 10 W COB LED with maximum emission at 705 nm and long pass filter RG695. There was practically no overlap between the lamp spectrum and the absorption of TMPyP. For experimental verification, we assessed the effect of red light exposure for 60 min on bacteria using 2 and 20 μm of the PS TMPyP in phosphate-buffered saline (PBS) spiked with 10^6^ CFU/mL *E. coli*. As illustrated in Supplementary Figure S1, the ambient red light showed no bactericidal effect within 60 min of irradiation using 2 μm TMPyP (10 mL in a Petri dish 94/16), even when placing the samples directly below the lamp at a distance of about 40 cm. The same result was obtained for 20 μM TMPyP. To verify the absence of ambient red light effects on spermatozoa, semen of seven boars extended in Androstar Premium (APrem) containing 2 μM TMPyP was exposed for 30 min to the dark room lamp and then stored up to 144 h at 17°C in the dark. Sperm motility assessed with computer-assisted semen analysis (Section 2.7.1) and membrane integrity assessed by flow cytometry (Section 2.7.2) did not differ from dark control samples (Supplementary Figure S2). Thus, the dark room lamp allowed visually controlled handling of the samples in red ambient light without harming the spermatozoa or accidentally activating the PS TMPyP.

### 2.3 Experimental design

**Figure 4:**
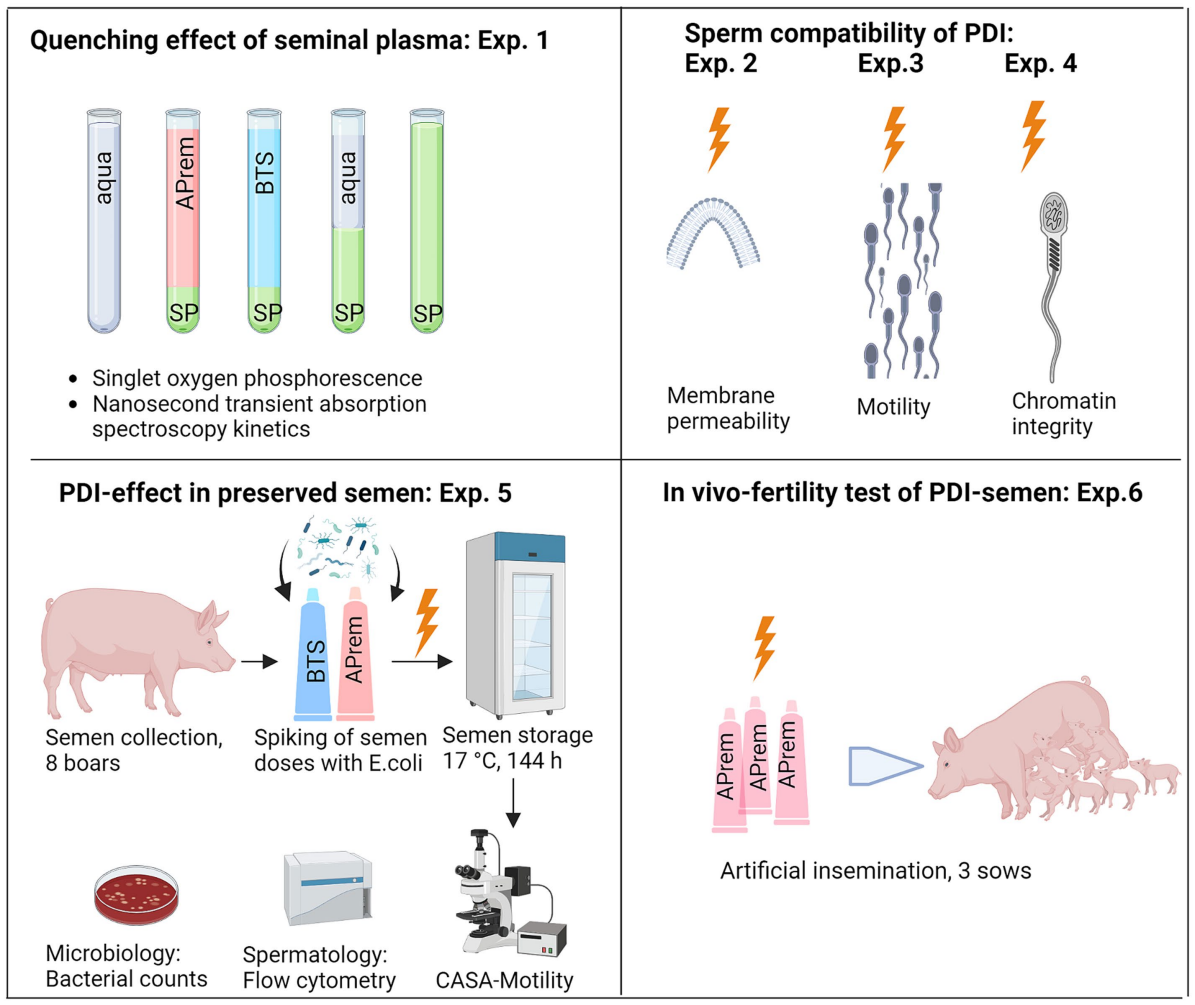
Overview of the experimental design, Experiments (Exp.) 1–6. SP: Seminal plasma; APrem: Androstar Premium semen extender; BTS: Beltsville Thawing Solution extender; PDI: Photodynamic inactivation, shown as orange lightning symbol; CASA: Computer-assisted semen analysis (Created with BioRender.com).

A series of six experiments was performed in extended semen samples, as demonstrated in Figure 4. Experiment 1 determined the quenching effect of seminal plasma in different concentrations and solutions for identifying the ideal sample type for the PDI. In Experiments 2–4, the sperm compatibility of the photodynamic treatment was examined using sperm motility, viability, and chromatin integrity as indicative parameters. After selection of a PDI method that caused minimal sperm damage and had high efficiency against bacteria (Supplementary Figure S3), Experiment 5 was designed to evaluate the PDI efficiency on bacteria and effects on sperm quality during long-term storage of semen in two different extenders, the short-term extender Beltsville Thawing Solution (BTS) and the cell-protective long-term extender APrem. Experiment 6 served as a proof-of-principle study, designed to verify whether sperm subjected to photodynamic treatment maintain their fertilizing capacity *in vivo*. The TMPyP concentrations and illumination intensities used in our experiments are shown in Table 2.

**Table 2:** Photosensitizer (TMPyP) concentration and illumination with white LED light used in the spermatology experiments.

	Experiment No.					
	1. Singlet oxygen kinetics	2. Membrane permeability	3. Motility effects	4. Chromatin integrity	5. Semen storage	6. *In vivo* fertility
TMPyP (μM)	0.5	5	2; 5	2	2	2
Light dose (mW/cm^2^)	9.5	9.5	9.5	9.5	4.5	4.5
Illumination time	3 min	30 min	3 × 3 min	30 min	90 s	90 s

#### 2.3.1 Experiment 1: Singlet oxygen phosphorescence and PS triplet kinetics

To examine the potential quenching effects of different solutions surrounding the spermatozoa, singlet oxygen phosphorescence kinetics of TMPyP in SP and its different aqueous solutions were examined. The setup comprised a TCMPC 1270 (SHB Analytics GmbH, Berlin, Germany), which consists of an improved version of the photomultiplier H10330 from Hamamatsu Photonics K.K., Hamamatsu, Japan, high etendue bandpass optics around 1,270 nm and a multiphoton counting electronics (12 kHz, 20 ns channel width), which guarantees linearity, even at high excitation intensities.

A power wand from Coherent Corp., Germany was used for all intensity measurements. Although the detector is well calibrated, for white light detection the wavelength sensitivity correction had to be back engineered from the internal correction factors.

The PS triplet time of TMPyP was determined by nanosecond transient absorption (nTAS). Our miniaturized setup included a parallel test light path using a microscope objective and an optically flat polished 5 mm LED. This high-quality light beam allows double discrimination of fluorescent emission from the sample by focusing through an aperture that matches the geometrical spot of the test light and bandpass filtering afterwards, before the signal is detected by a fast photodiode with low noise amplifier (Elektronik Manufaktur Mahlsdorf, Germany). The test wavelength is selected to test induced absorption from the T_1_ state to higher triplet states, hence it allows direct determination of the PS triplet decay.

#### 2.3.2 Experiment 2: Sperm membrane permeability of TMPyP

Semen samples from different boars (*n* = 2) extended in BTS with 5 μM TMPyP were incubated for 1 h in the dark before the same samples were illuminated with a light intensity of 9.5 mW/cm^2^ for 30 min. As a positive control, a sperm suspension incubated in the dark was frozen at −20°C for 20 min to disrupt the membrane integrity.

#### 2.3.3 Experiment 3: Dark and light effects of TMPyP on sperm motility

Semen samples from different boars (*n* = 3) extended in BTS with different concentrations of TMPyP were incubated in the dark or exposed to 3 cycles with 3 min illumination and 5 min darkness in between. Sperm motility (total) was assessed with computer-assisted semen analysis (Section 2.7.1).

#### 2.3.4 Experiment 4: Effect of PDI on sperm chromatin integrity

A pooled semen sample from six boars was extended in BTS with 2 μM TMPyP. Aliquots were kept in the dark (control) or exposed to 9.5 mW/cm^2^ for 30 min (PDI). DNA integrity was evaluated in 1,000 spermatozoa per aliquot using the sperm chromatin dispersion assay (Section 2.7.3).

#### 2.3.5 Experiment 5: Effect of PDI on bacterial load and sperm quality in stored semen

Semen samples (*n* = 8) were extended in the short-term extender BTS and the long-term extender Androstar Premium (APrem), both supplemented with 2 μM TMPyP. The extenders were spiked with *Escherichia coli* to enhance the bacterial load in the semen samples. Samples were subjected to PDI with 5 mW/cm^2^ for 90 s. At 5 h, 72 h, and 144 h of semen storage, bacterial counts were determined (Section 2.6). Additionally, sperm kinematics were recorded by computer-assisted semen analysis (Section 2.7.1), and sperm membrane integrity and mitochondrial activity were evaluated by flow cytometry (Section 2.7.2).

#### 2.3.6 Experiment 6: *In vivo* fertility of PDI-semen

Semen samples subjected to PDI were tested for their ability to fertilize *in vivo*. The experiment served as proof-of-principle to exclude that the photodynamic treatment impairs essential functions of spermatozoa, which may have remained undetected by the *in vitro* tests. Three sows housed at the teaching and research field station Ruthe of the University of Veterinary Medicine Hannover, Hannover, Germany were inseminated. The sows are part of the university-owned breeding herd and were inseminated in spontaneous estrus by trained personnel in the farm’s routine breeding program. One normospermic boar ejaculate was extended in APrem to 25 × 10^6^ sperm/mL without conventional antibiotics and subjected to PDI as described above by using 2 μM TMPyP and illumination with 5 mW/cm^2^ for 90 s. After PDI, the semen was filled into semen tubes (QuickTip Flexitube, 95 mL, Minitüb GmbH), each resulting in 2 × 10^9^ sperm in a volume of 80 mL extended semen. The semen tubes (*n* = 10) were stored in the dark at 17°C and transported to the sow farm. Sows were checked for estrus with a teaser boar twice daily. The day after semen collection (24 h), sows were first inseminated followed by a second insemination on the following day. Sows were checked for return to estrus 18–22 days after insemination, and pregnancy control was performed by real-time ultrasound at d 28 after insemination. Litter sizes were recorded as the number of total born piglets and live born piglets.

### 2.4 Chemicals and semen extenders

Chemicals of analytical grade were purchased from Sigma-Aldrich Productions GmbH (Steinheim, Germany), Carl Roth GmbH & Co. KG (Karlsruhe, Germany), Merck KGaA (Darmstadt, Germany), BIOZOL Diagnostica Vertrieb GmbH, Eching, Germany, Biomol GmbH, Hamburg, Germany. TMPyP was purchased from TCI Chemicals (Tokyo, Japan). Semen extenders were obtained from Minitüb GmbH (Tiefenbach, Germany). The BTS extender consists of 205 mM glucose, 20.4 mM Na_3_C_6_H_5_O_7_, 10.0 mM KCl, 15 mM NaHCO_3_, and 3.36 mM EDTA (Johnson et al., 1988). The APrem extender containing a cell shield protecting component and an organic bactericidal supplement is designed for long-term semen storage [Minitüb GmbH; Ref. 13533/7001; (Minitüb, 2024)]. Semen extenders were free of conventional antibiotics and were sterile filtered before use.

### 2.5 Semen collection and processing

Semen was collected from eight sexually matured, healthy boars housed on straw in individual pens at the Unit for Reproductive Medicine, University of Veterinary Medicine Hannover. The boars of four different breeds (Piétrain, Landrace, Duroc, Large White), 1 to 5 years old, were treated in accordance with the European Commission Directive for Pig Welfare following the ARRIVE guidelines. At weekly intervals, entire ejaculates without the bulbourethral gland secretion were routinely collected by trained technicians using the gloved hand method. All procedures involving animals were approved by the Animal Welfare Committee of the University of Veterinary Medicine Hannover. The ejaculates were normospermic and fulfilled the standards for semen use in artificial insemination. These comprised at least 70% motile spermatozoa and a maximum of 25% morphological abnormal sperm (BRS, 2023). The raw semen was extended with pre-warmed (35°C) BTS or APrem extender to 20 × 10^6^ sperm/mL at a final volume of 100 mL. The extended semen was kept at room temperature for 90 min and then stored at 17°C in the dark. Sample bags with 8 mL extended semen were then prepared as described in Section 2.1. All experiments were performed in a darkened room at room temperature. For Experiment 1, sperm-free seminal plasma was collected from raw semen by two centrifugations at 3,360*g* for 10 min.

### 2.6 Bacterial inoculation of samples and bacterial count

The raw semen contained between 2.0 × 10^2^ and 1.1 × 10^4^ CFU/mL (Table 3), among these five gram-negative bacterial species belonging to the order Enterobacterales (*n* = 4) and Pseudomonadales (*n* = 1) as well as two gram-positive bacterial species belonging to the order Bacillales and Mycobacteriales. Dilution of the raw semen reduced the bacterial count by approximately one log level. Extended semen samples were spiked with *E. coli* to enhance the bacterial load to approximately 5 × 10^3^ CFU/mL. The *E. coli* bacteria were isolated from boar semen and the bacterial species was determined by MALDI-TOF MS (microFlex LT, Bruker Daltonics GmbH & Co. KG, Bremen, Germany) with the software Biotyper (Bruker Daltonics GmbH & Co. KG, Server Version 4.1.100). Bacterial isolates were stored at −80°C, and then cultured on sheep blood agar for 24 h at 35°C under aerobic conditions. The bacterial counts were determined from 10-fold serial dilutions in PBS ranging from 10^−1^ to 10^−10^. A volume of 100 μL of each dilution was plated on sheep blood agar and incubated for 24 h at 35°C under aerobic conditions. Bacterial colonies were counted, and total bacterial numbers were calculated and expressed as CFU/mL.

**Table 3:** Bacterial counts and bacterial species isolated from raw semen (*n* = 14 ejaculates of eight boars).

	Min	Max	Mean	SD
Bacterial count (CFU/mL)	2.0 × 10^2^	1.1 × 10^4^	4.1 × 10^3^	3.4 × 10^3^

	Bacterial species
Gram-negative	*Escherichia coli; Pseudomonas aeruginosa; Providencia stuartii; Citrobacter koseri; Proteus vulgaris*
Gram-positive	*Staphylococcus species; Corynebacterium freneyi*

### 2.7 Spermatology

#### 2.7.1 Computer-assisted semen analysis

Sperm kinematics were assessed with the computer-assisted semen analysis (CASA) system AndroVision^®^ (Version 1.2, Minitüb GmbH) equipped with a TV adapter (60-C 1″ 1.0×, Carl Zeiss Microscopy GmbH, Jena, Germany), a digital camera (acA2440–75uc, Basler AG, Ahrensburg, Germany), and a heated automatic scan stage (Höfner et al., 2020). Aliquots of 2 mL extended semen were prewarmed under air at 38°C for 30 min in a water bath before filling a 20 μm “Leja” counting chamber (Leja Products, B.V., Nieuw Vennep, The Netherlands). At least 500 spermatozoa were recorded at 100× magnification with a rate of 75 pictures per s. A spermatozoon was considered “motile” when its curved-line velocity (VCL) was higher than 24 μm/s and its amplitude of lateral head displacement was higher than 1 μm. Progressively motile spermatozoa were determined by a VCL higher than 41 μm/s and a velocity straight line (VSL) higher than 15 μm/s. The kinematic parameters total motility (%), progressive motility (%), VCL (μm/s), amplitude of lateral head displacement (ALH; μm), and beat cross frequency (BCF; Hz) were determined (Boyers et al., 1989).

#### 2.7.2 Flow cytometry

A flow cytometer (Cyto Flex, Beckman Coulter GmbH, Krefeld, Germany) equipped with three lasers (488 nm, 50 mW, 638 nm, 50 mW, 405 nm, 80 mW) was used. The gating was performed with CytExpert 2.4 Software (Beckman Coulter GmbH).

The integrity of the plasma membrane and acrosome were evaluated as described previously (Höfner et al., 2020) with some modification in the samples containing TMPyP. Briefly, semen samples were stained with final concentrations of 1.3 μM Hoechst (H) 33342, 1.5 μM propidium iodide (PI), and 2 μM fluorescein conjugated peanut agglutinin (FITC-PNA). The photosensitizer TMPyP is, like PI, a membrane-impermeable DNA stain with a similar emission spectrum to PI. For this reason, PI was omitted in samples containing TMPyP. In these samples, final concentrations were 100 μM TMPyP, 1.3 μM H 33342, and 2 μM FITC-PNA. Hoechst 33342 was detected on fluorescence detector PB-450 (450/45 nm BP), FITC-PNA on FITC (525/40 nm BP), PI on PC5.5 (690/50 nm BP), and TMPyP on BV650 (660 nm/20 BP). Non-DNA containing particles were identified by negative H 33342 stain and were excluded from analysis. At least 10,000 individual spermatozoa per sample were evaluated. Spermatozoa with intact membranes were negative for the stains of PI and FITC-PNA.

The mitochondrial activity was assessed as described previously (Schulze et al., 2013) with some modifications. Briefly, semen aliquots of 50 μL were incubated for 20 min at 38°C in 950 μL HBS containing final concentrations of 2.7 μM H 33342, 0.003 μM rhodamine (Rh) 123, 0.19 mmol/L PI. Rhodamine was detected on FITC (525/40 nm BP). Viable sperm with mitochondrial activity were negative for the PI stain and positive for Rh123.

#### 2.7.3 Sperm chromatin dispersion assay

DNA damage in spermatozoa was assessed using the sperm chromatin dispersion test, as previously described (Fernández et al., 2003; Oldenhof et al., 2017) with some modifications. Extended (non-frozen) semen samples were washed twice with PBS (pH 7.4). Thereafter, 25 μL resuspended semen containing 50 × 10^6^ sperm/ mL was diluted in 800 μL of 1% low-melt agarose (w/v in PBS, pH 7.4) at 37°C. Two 14 μL sperm/agarose-samples were added per agarose-coated slide and each directly covered with coverslips (18 × 18 mm), followed by solidification for 5 min at 4°C and subsequent removal of the coverslips. Slides with spermatozoa embedded in agarose were then incubated at room temperature for 12 min with acid solution (0.08 N HCl), followed by 40 min incubation in freshly prepared lysis solution (2.5 mM NaCl, 0.1 M Na_2_EDTA, 10 mM TRIS, 0.1% Triton-X100, 25 mM DTT). Thereafter, slides were washed in distilled water for 2 min, and specimens were dehydrated through a graded ethanol series (70, 90, and 100% ethanol, 2 min each). After air-drying, specimens were stained for 2 min with 1 mL Wright’s eosin-methylene blue solution (Carl Roth GmbH & Co. KG, Karlsruhe, Germany), an equal volume of PBS (pH 6.8) was added for further staining during 15 min, followed by washing under tap water and air-drying. Slides were examined using a light microscope (BX60; Olympus Europa SE & Co. KG, Hamburg, Germany), at 400× magnification, and a minimum of 1,000 sperm per sample were analyzed for the presence of DNA dispersion halos around the sperm head. After exposure to acid and lysis solutions, spermatozoa with intact chromatin exhibit dispersed nuclei and a typical halo around the sperm head, whereas such a halo formation is absent in sperm with damaged/fragmented DNA (Fernández et al., 2003).

### 2.8 Statistical analysis

Data were analyzed with IBM SPSS Statistics Professional (IBM Corp., Armonk, NY, United States). Data were checked for normal distribution using the Kolmogorov–Smirnov test and the Shapiro–Wilk test. To address the repeated measurements of microbiology data, the Friedman test (XLSX) or Kruskal Wallis test (XLSX) was performed. Pairwise comparisons were performed with the Wilcoxon test and corrected by Holm Bonferroni. Spermatology data were compared between treatments using a paired Student t-test or two-way ANOVA with repeated measures and a Bonferroni *post hoc* test. A *p*-value of less than 0.05 was considered statistically significant. Unless otherwise stated, data are presented as mean ± standard error of the mean (SEM).

## 3 Results

### 3.1 Experiment 1: Singlet oxygen phosphorescence and PS triplet kinetics

**Figure 5:**
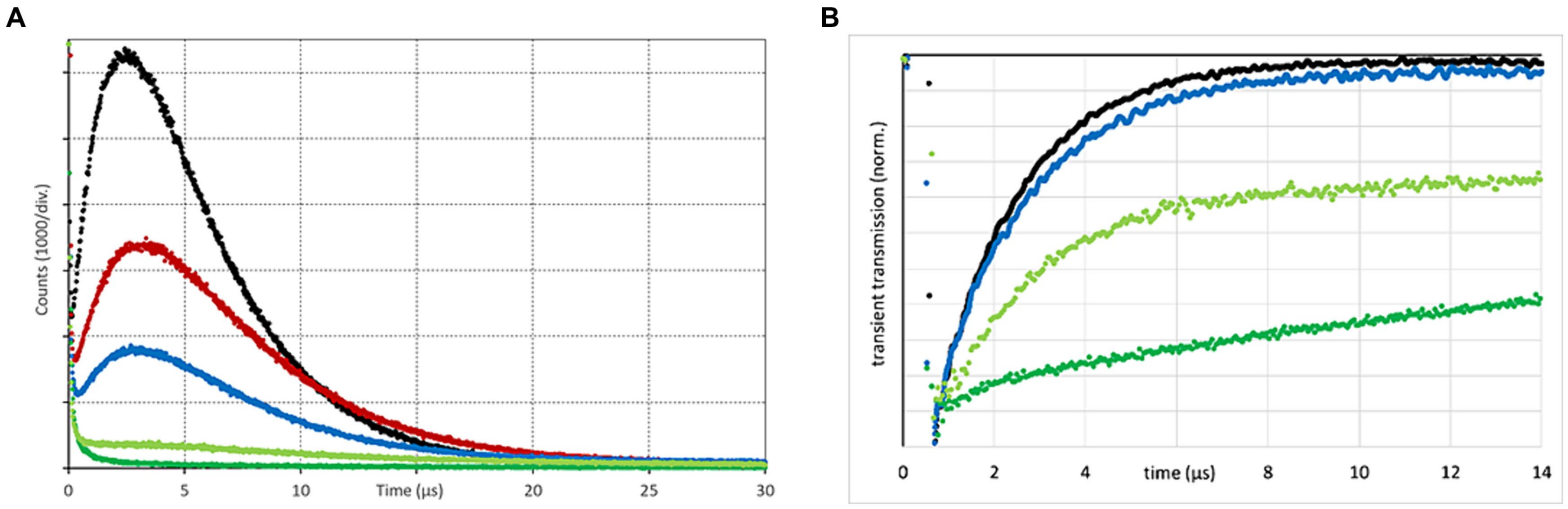
Singlet oxygen phosphorescence kinetics **(A)**, as determined for TMPyP in water (black), 10% (v/v) seminal plasma (SP) in Androstar Premium (red), 10% (v/v) SP in Beltsville Thawing Solution (blue), 50% (v/v) SP in water (light green), and pure seminal plasma (dark green). In the presence of SP, the TMPyP has a tendency to bleach under intense illumination. The curves shown here for the two dilutions in extenders are therefore averaged signals before and after illumination with 9.5 mW/cm^2^ over 3 min. Nanosecond transient absorption spectroscopy kinetics **(B)** using the same color indication; Experiment 1.

To examine potential quenching effects of different solutions surrounding the spermatozoa, singlet oxygen phosphorescence kinetics of TMPyP in SP and different aqueous solutions thereof were recorded and compared to that in pure water (Figure 5A). Tested SP concentrations were 100, 50% (v/v) in water and 10% (v/v) in the two semen extenders BTS and APrem before and after illumination.

Kinetics show that SP acts as a highly efficient static quencher, which is still the case after dilution (50%) in water. Higher dilution in extenders reduced that effect. The extender APrem yielded a higher ^1^O_2_ generation compared to BTS. Comparison of ^1^O_2_ generation in water indicates the presence of some static quenching (shielding) for extenders. An interesting observation is that the ^1^O_2_ signal was reduced to a higher extent than the nanosecond transient absorption spectroscopy (ns-TAS) kinetics for similar samples (shown in Figure 5B) can explain. The reason for this difference is not yet clear. The PS triplet time of TMPyP as determined by nanosecond transient absorption spectroscopy (nsTAS) in water was 1.9 ± 0.1 μs, which is a typical value for water soluble tetrapyrroles. Adding 10% SP, the triplet decay time slightly increased up to 2.0 ± 0.1 μs and a small percentage of the signal decayed much slower, reflecting some static shielding effect. Increasing the SP amount to 50% resulted in a double exponential decay with 2.2 ± 0.1 μs and > 100 μs. The data clearly suggest that SP has a certain capability to “capture” TMPyP, some sort of static interaction between the TMPyP and SP that reduces the accessibility of oxygen to the excited PS and thus prevents ^1^O_2_ generation. Dilution of the SP down to 10% or less did not completely eradicate this effect but reduced it to an acceptable percentage. For this reason, in the following spermatology experiments, the PDI was not applied in raw semen containing ~97% (v/v) SP but in extended semen doses as used for artificial insemination, usually containing around 10% (v/v) SP.

### 3.2 Experiment 2: Sperm membrane permeability of TMPyP

**Figure 6:**
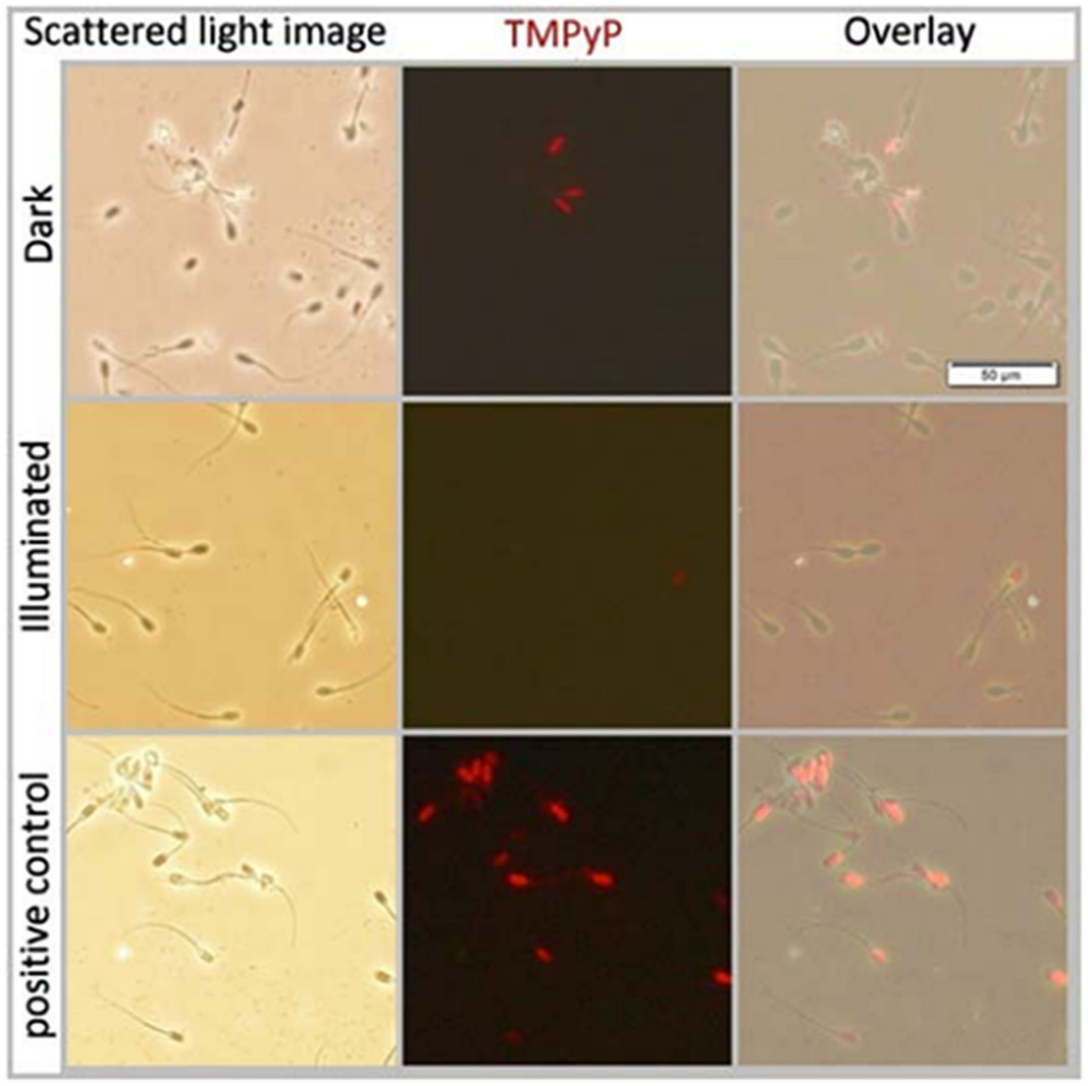
Scattered light- and fluorescence microscopy images and their overlays of boar spermatozoa extended in Beltsville Thawing Solution. Samples contained 5 μM TMPyP and were incubated for 1 h in the dark (top row) before illumination using white LED light with 9.5 mW/cm^2^ for 30 min (middle row). As a positive control to test membrane permeability for TMPyP, a sperm suspension incubated in the dark was frozen at −20°C for 20 min to disrupt the membrane integrity; Experiment 2.

Co-incubation of extended semen with 5 μM TMPyP showed that TMPyP did not penetrate the plasma membranes of viable (= plasma membrane-intact) spermatozoa, either in the dark or after exposition to 9.5 mW/cm^2^ for 30 min. When sperm membranes were disrupted by shock-freezing (positive controls), all spermatozoa were stained positively for the DNA-stain TMPyP (Figure 6).

### 3.3 Experiment 3: Dark and light effects of TMPyP on sperm motility

**Figure 7:**
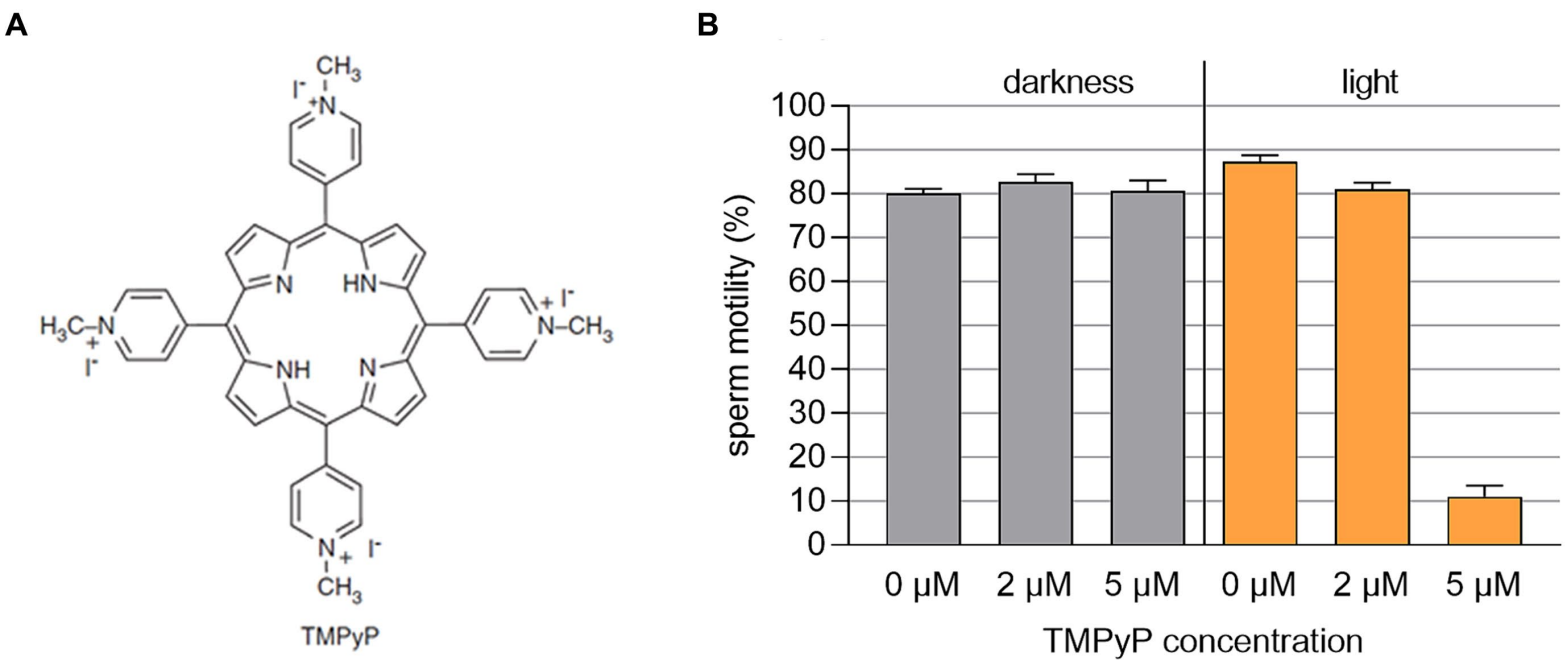
**(A)** Structural chemical formula of the photosensitizer TMPyP. **(B)** Sperm motility in boar semen samples extended with Beltsville Thawing Solution containing different concentrations of TMPyP. Samples were kept in the dark or illuminated using white LED light in 3 cycles with 3 min illumination at 9.5 mW/cm^2^ followed by 5 min darkness, *n* = 3 samples from different boars; Experiment 3.

The TMPyP in a concentration up to 5 μM showed no dark toxicity, whereas exposure of the semen samples containing 5 μM TMPyP to three illumination cycles with 9.5 mW/cm^2^ resulted in drastic loss of motility (Figure 7).

### 3.4 Experiment 4: Effect of PDI on sperm chromatin integrity

**Figure 8:**
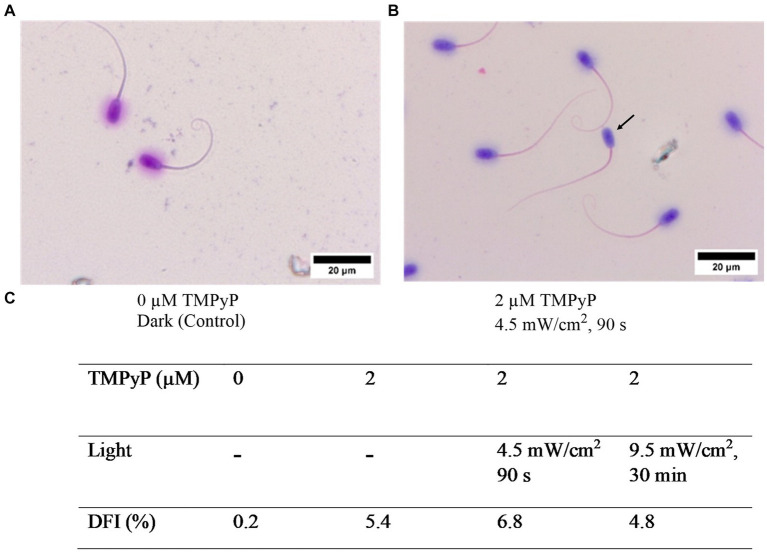
Effect of the TMPyP and illumination on sperm chromatin integrity evaluated with the sperm chromatin dispersion assay. **(A)** Control sample. **(B)** After illumination in presence of TMPyP. Intact DNA is visualized by a halo around the sperm head. Arrow: Sperm lacking a halo indicating fragmented DNA. **(C)** DNA fragmentation index (DFI) obtained after photodynamic treatment of semen samples using different TMPyP concentration and illuminations. Pooled semen samples of 6 boars, *n* = 1,000 spermatozoa per sample; Experiment 4.

Results of the sperm chromatin dispersion assay revealed no effect on sperm DNA integrity with 2 μM TMPyP and illumination with 9.5 mW/cm^2^ for 30 min (Figure 8).

### 3.5 Experiment 5: Effect of PDI on bacterial growth and sperm quality in stored semen

Pre-experiments with semen extended in the simple short-term extender BTS medium showed that a PDI with 2 μM TMPyP and a white LED light intensity of 5.0 mW/cm^2^ had relatively high antimicrobial effect and a low impact on sperm kinematics after long-term storage for 144 h (Supplementary Figure S3; Supplementary Table S1). This PDI approach was then used in the following experiments to test whether the antimicrobial efficiency and the sperm compatibility could be increased by using the sperm protective long-term extender APrem.

#### 3.5.1 Bacterial growth

**Figure 9:**
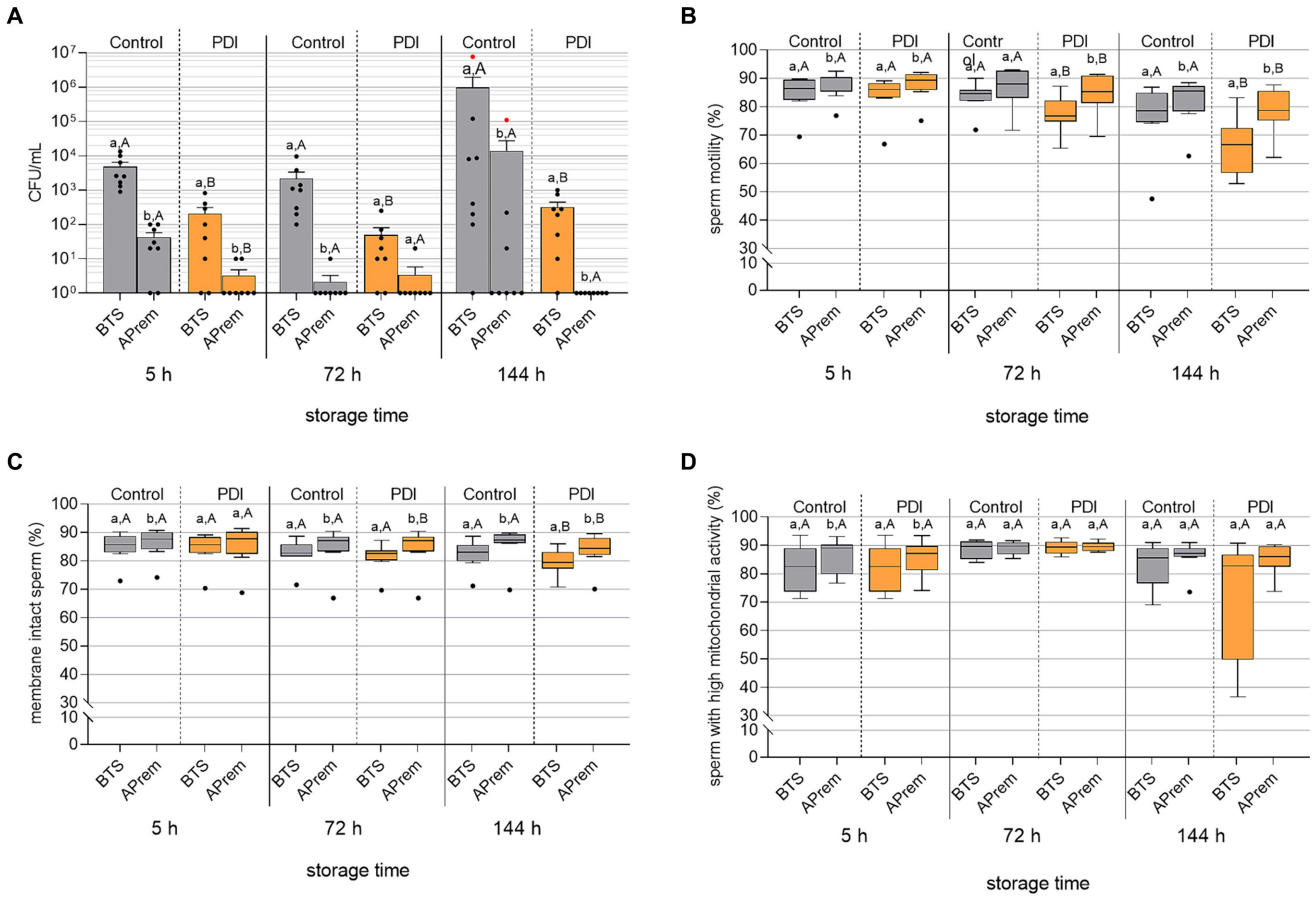
Effect of Photodynamic Inactivation (PDI) on bacterial counts [CFU/mL; **(A)**], sperm motility **(B)**, sperm membrane integrity **(C)** and mitochondrial activity in viable sperm **(D)** in extended boar semen (*n* = 8 different boars) during long-term storage at 17°C. Semen was extended in Beltsville Thawing Solution (BTS) and Androstar Premium (APrem), both containing 2 μM TMPyP and spiked with 5 × 10^3^ CFU/mL *E. coli*. PDI in extended semen: white LED light, 4.5 mW/cm^2^, 90 s. Red dots in **(A)**: Samples contained *Proteus vulgaris* in pure culture. a,b: Different lowercase letters indicate differences between extenders within storage times and treatment (*p* < 0.05). A,B: Different uppercase letters indicate differences between PDI and control within storage time and extender (*p* < 0.05); Experiment 5.

Results of bacterial growth during 144 h of semen storage are shown in Figure 9A. The PDI reduced the bacterial counts to less than 10^3^ CFU/mL in all semen samples within five hours after dilution and treatment. At all timepoints, the bacterial counts were lower in samples extended in APrem compared to BTS. At 144 h in the APrem samples, bacterial counts were below the detection limit (< 10 CFU/mL), whereas bacterial counts in control samples increased to ~10^4^ CFU/mL in APrem and ~ 10^6^ CFU/mL in the BTS samples. In the control samples of both extenders, *Proteus vulgaris* was identified as the dominating bacteria species. *Proteus vulgaris* was not detected after PDI.

#### 3.5.2 Sperm kinematics

Results of sperm kinematic assessment during 144 h of semen storage are shown in Figure 9B and Table 4. The PDI had an effect on motility in all semen samples at 72 h and 144 h of storage. PDI samples extended in APrem had higher proportions of motile sperm compared to BTS at all time points. At 144 h, the percentage of motile sperm in the PDI samples extended in APrem was (78.3 ± 2.9%), and in samples extended in BTS 66.1 ± 3.6% (*p* < 0.05). At 5 h after semen extension and treatment, average values for sperm velocity (VCL) and the amplitude of lateral sperm head displacement (ALH) were higher in PDI samples compared to controls in both types of extenders. There were no PDI effects on VCL and ALH at 72 h or 144 h, and on the Beat Cross Frequency (BCF) of the sperm tail at all time points.

**Table 4:** Effect of photodynamic inactivation (PDI) on sperm kinematics in extended boar semen during long-term storage at 17°C.

Parameter	Extender and treatment	5 h storage	72 h storage	144 h storage
Progressive motility (%)	BTS, Control	74.2 ± 4.0^a,A^	66.9 ± 3.8^a,A^	63.8 ± 6.6^a,A^
	APrem, Control	84.3 ± 2.3^b,A^	78.7 ± 4.0^b,A^	72.0 ± 6.0^b,A^
	BTS, PDI	74.2 ± 4.2^a,A^	62.9 ± 3.7^a,A^	52.9 ± 5.5^a,B^
	APrem, PDI	84.4 ± 2.8^b,A^	76.7 ± 4.5^b,A^	65.7 ± 5.9^b,B^
Velocity curvilinear line (μm/s)	BTS, Control	134.6 ± 6.6^a,A^	136.2 ± 8.0^a,A^	130.8 ± 11.4^a,A^
	APrem, Control	169.7 ± 5.4^b,A^	159.3 ± 12.8^b,A^	153.3 ± 17.5^a,A^
	BTS, PDI	113.3 ± 6.2^a,B^	134.0 ± 8.8^a,A^	130.9 ± 10.4^a,A^
	APrem, PDI	158.2 ± 4.9^b,B^	160.8 ± 13.4^b,A^	152.4 ± 19.0^a,A^
Velocity straight line (μm/s)	BTS, Control	53.9 ± 3.2^a,A^	46.6 ± 4.4^a,A^	52.9 ± 5.9^a,A^
	APrem, Control	75.1 ± 3.1^b,A^	64.3 ± 6.5^b,A^	67.0 ± 8.7^a,A^
	BTS, PDI	55.8 ± 3.4^a,A^	49.6 ± 4.5^a,A^	48.0 ± 6.0^a,A^
	APrem, PDI	75.4 ± 2.9^b,A^	66.5 ± 7.1^b,A^	63.8 ± 8.5^b,A^
Velocity average path (μm/s)	BTS, Control	64.9 ± 3.8^a,A^	60.7 ± 5.3^a,A^	63.8 ± 6.7^a,A^
	APrem, Control	88.9 ± 3.5^b,A^	80.0 ± 7.7^b,A^	79.6 ± 10.1^a,A^
	BTS, PDI	63.2 ± 3.6^a,A^	64.8 ± 4.9^a,A^	60.8 ± 6.3^a,A^
	APrem, PDI	86.9 ± 3.1^b,A^	82.2 ± 8.3^b,A^	76.0 ± 10.0^b,B^
Amplitude of lateral head displacement (μm)	BTS, Control	1.15 ± 0.06^a,A^	1.20 ± 0.07^a,A^	1.13 ± 0.09^a,A^
	APrem, Control	1.37 ± 0.04^b,A^	1.34 ± 0.10^a,A^	1.29 ± 0.13^a,A^
	BTS, PDI	0.92 ± 0.05^a,B^	1.15 ± 0.08^a,A^	1.20 ± 0.09^a,A^
	APrem, PDI	1.26 ± 0.04^b,B^	1.34 ± 0.10^b,A^	1.35 ± 0.16^a,A^
Beat cross frequency (Hz)	BTS, Control	31.7 ± 0.8^a,A^	29.3 ± 1.0^a,A^	28.4 ± 1.2^a,A^
	APrem, Control	32.4 ± 0.7^a,A^	29.6 ± 0.7^a,A^	28.0 ± 0.9^a,A^
	BTS, PDI	32.0 ± 0.6^a,A^	29.8 ± 0.6^a,A^	26.9 ± 1.0^a,A^
	APrem, PDI	32.6 ± 0.7^a,A^	29.7 ± 0.8^a,A^	25.7 ± 1.0^a,B^

Semen was extended in Beltsville Thawing Solution (BTS) and Androstar Premium (APrem), both containing 2 μM TMPyP, and spiked with 5 × 10^3^ CFU/mL *E. coli*. PDI in extended semen: white LED light, 4.5 mW/cm^2^, 90 s. a,b: Different lowercase letters indicate differences between extenders within storage times and treatment (*p* < 0.05). A,B: Different uppercase letters indicate differences between PDI and control within storage time and extender (*p* < 0.05). Data are means and SEM (*n* = 8); Experiment 5.

#### 3.5.3 Sperm membrane integrity

The integrity of the sperm plasma membranes and acrosomes is shown in Figure 9C. The effect of the PDI on sperm membrane integrity was small and became visible at 72 h of storage. At all timepoints, PDI samples extended in APrem had higher percentages of membrane-intact spermatozoa compared to BTS. At 144 h, the percentage of membrane-intact spermatozoa was 83.7 ± 2.2% for PDI samples extended in APrem, and 79.4 ± 1.6% for samples extended in BTS (*p* < 0.05).

#### 3.5.4 Mitochondrial activity

Results for mitochondrial activity in viable spermatozoa are shown in Figure 9D. In both extenders and at all time points, there was no effect of the PDI on the mitochondrial activity of viable spermatozoa; there was a higher variation at 144 h in the BTS samples compared to the APrem samples. At 144 h, the percentage of viable spermatozoa with high mitochondrial activity was 85.2 ± 1.9% in the APrem extender and 72.6 ± 7.5% in the BTS extender (*p* > 0.05).

### 3.6 Experiment 6: *In vivo* fertility of PDI-semen

**Figure 10:**
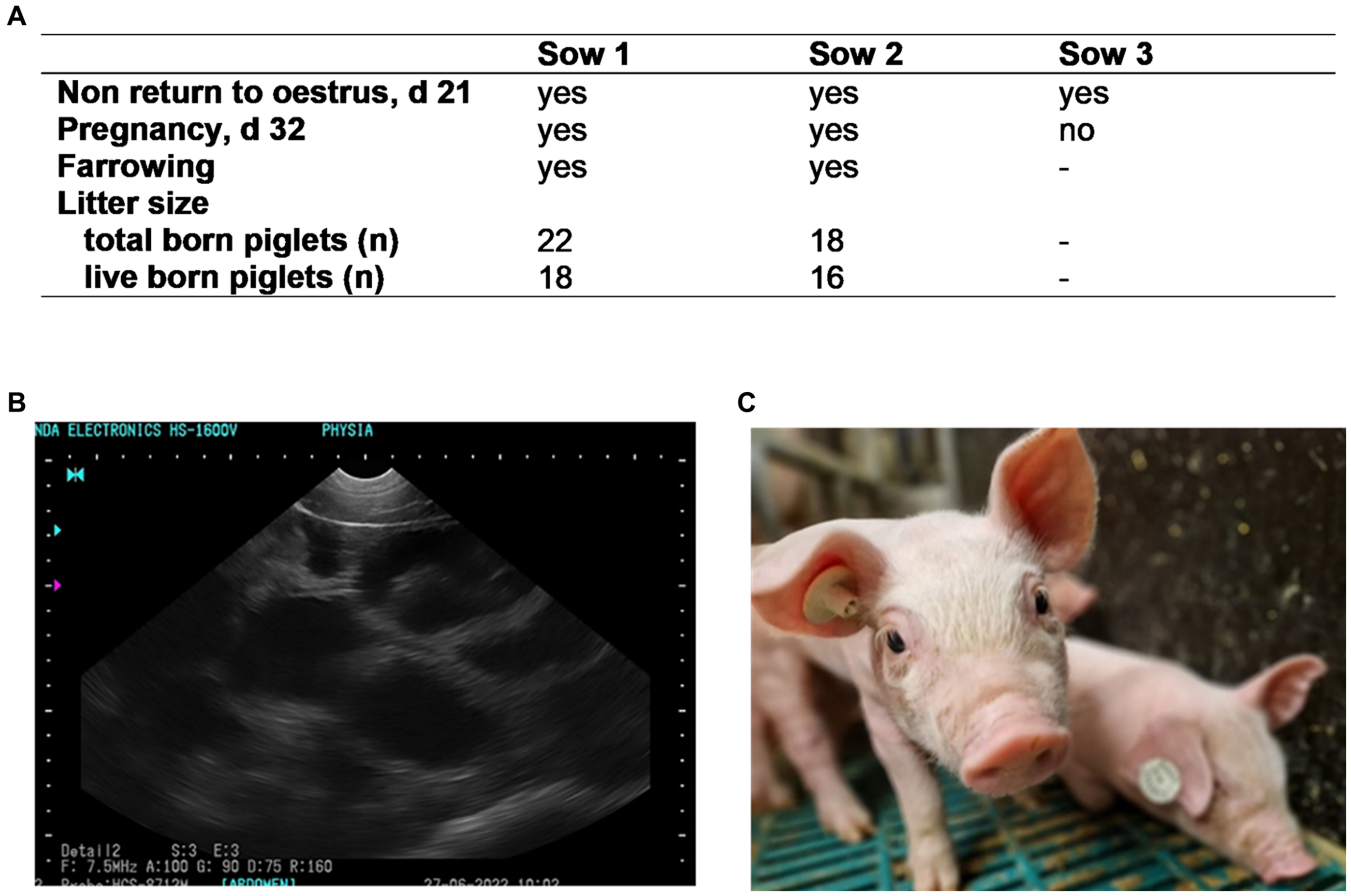
Fertility **(A)** of three sows inseminated with semen subjected to Photodynamic inactivation (PDI) of bacteria. Semen from one boar was extended in Androstar Premium (APrem) containing 2 μM TMPyP. PDI: white LED light, 4.5 mW/cm^2^, 90 s. **(B)** Positive pregnancy control at d 32 (black spots); (**C**): live born, healthy piglet obtained from PDI semen; Experiment 6.

At 48 h, sperm motility of the semen used for insemination was 84.1%, the percentage of membrane intact spermatozoa was 88.0%, and the bacterial count was 10 CFU/mL. Results of the *in vivo* fertilization trial are presented in Figure 10. None of the three inseminated sows returned to estrus within 21 d after insemination, indicating that they all conceived. At d 32, pregnancy was confirmed in two sows by ultrasound, resulting in a high number of healthy piglets.

## 4 Discussion

The present study shows that PDI is an innovative tool to inactivate bacteria in extended boar semen while maintaining sperm fertility. We chose porphyrin TMPyP as an eco-friendly and non-hazardous photosensitive molecule, which is well-known for being an efficient PDI drug against gram-positive and gram-negative bacteria (Nitzan et al., 1995; Preuß et al., 2016). Importantly, it is not harmful to somatic mammalian cells after illumination (Pohl et al., 2016).

The positively charged dye molecules are attracted by the negatively charged cell walls (Nitzan et al., 1995; Alves et al., 2009; Eckl et al., 2018). Sperm outer membranes, similar to bacterial cell walls, are negatively charged and therefore, are also targeted by cationic PS. However, as shown here, TMPyP is not spermicidal. Importantly, even at illumination for 30 min, TMPyP is not taken up by the spermatozoa and does not affect sperm viability. This minimizes the risk of detrimental effects on the sperm, including impairment mitochondrial activity, and prevents the PS from interfering with the sperm DNA.

Studies with confocal laser scanning microscopy and fluorescence lifetime imaging revealed that bacteria can be efficiently inactivated without intracellular uptake of TMPyP (Preuss et al., 2013). This is especially interesting because the PS activity outside of the bacterial cell further diminishes the risk of the development of resistance to the photodynamic approach, which, in general, is regarded as low (Tavares et al., 2010). The phototoxic effect without PS entry into the cell was shown with *E. coli* (Preuss et al., 2013), a gram-negative bacterium that is also frequently detected in boar semen (Althouse and Lu, 2005; Luther et al., 2023). Gram-negative bacteria, which dominate the bacterial population in semen, are more difficult to target by the PDI compared to gram-positive bacteria due to their more complex cell walls. The present study clearly shows that PDI is efficient for the eradication of gram-negative and gram-positive bacteria typically present in raw boar semen. Collected raw semen is not a sterile fluid, as confirmed here by the presence of a mixed population of gram-negative and some gram-positive bacterial species. For challenging the experimental system with enhanced bacterial counts, extended semen samples were additionally spiked with the isolate *E. coli*, which is one of the predominant contaminants in boar semen doses (Althouse et al., 2000). The PDI diminished the bacterial counts to <10 CFU/mL in the preserved semen, thus proving efficiency against the typical commensal bacterial flora present in semen. It is noteworthy that during the one week of storage at 17°C, bacterial counts remained low, indicating lasting photodynamic growth inhibition in a semen extender milieu. Further studies should include other opportunistic pathogenic bacteria detected in boar semen, including environmental multi-resistant bacteria, such as *Serratia marcescens* and *Klebsiella oxytoca* (Costinar et al., 2021).

Notwithstanding, our study demonstrates that the PDI using TMPyP selectively inactivates seminal bacteria without affecting the essential functionality of sperm. Spermatozoa may be protected by their endogenous antioxidant defense systems that balance redox homeostasis, such as superoxide dismutase (Orzołek et al., 2013) or glutathione synthesis (Zhu et al., 2022). The different PDI effects on bacterial and sperm cells could also be explained by the distinct cell dimensions and the short free diffusion length of ^1^O_2_. At PS concentrations of 2 μM, the average distance between evenly distributed PS is only around 0.1 μm. Given the short PS triplet decay time (3.6 ± 0.1 μs in pure water), the diffusion length of ^1^O_2_ is less than 1 μm (Hackbarth et al., 2016). Quenching of ^1^O_2_ effects in biological systems, as also shown here for seminal plasma, further reduces the ^1^O_2_ decay time by up to a factor of 10 and thus the diffusion length by a factor of three (Kanofsky, 2011; Hackbarth et al., 2012). With no or low positioning selectivity of the PS, both bacteria and sperm will likely be within the diffusion length of the produced ^1^O_2_. However, given the small cell size of bacteria [*E. coli*: 2.0 μm long, 0.5 μm wide; (Levin and Angert, 2015)] compared to boar spermatozoa [head: 9 μm long, 5 μm wide; total length: 55 μm; (Wysokińska and Kondracki, 2019)], bacterial cells might be fully attacked by the short presence of ^1^O_2_,whereas the spermatozoa, possessing a larger cell volume, are less affected.

The quenching effect in a given solute can be visualized by ^1^O_2_ phosphorescence kinetics, which are determined by two decay times: the PS triplet decay and the singlet oxygen decay (Hackbarth et al., 2021). Here, we show that the environment of spermatozoa had a profound impact. Seminal plasma, a fluid rich in proteins, acts as a highly efficient static quencher, thereby preventing the production of ^1^O_2._ For this reason, the use of the PDI principle will not be successful in raw semen. The dilution of the SP down to 10% or less in semen extender media, as commonly done in semen preservation, does not completely eradicate the quenching effect, but reduces it to an acceptable level. When comparing the standard extender BTS with APrem from the standpoint of ^1^O_2_ generation, APrem is more efficient. The static quenching (shielding) is less pronounced in APrem. The reason for this is not yet clear. It may be that APrem acts against the shielding effect of SP components by competing with the PS or by competing with SP by being attractive for the PS itself.

The higher PDI efficiency when using APrem compared to BTS is mirrored in higher activity against bacteria during long-term semen preservation. An additional advantage of using the long-term extender APrem over the simpler short-term extender BTS is better protection against PDI-induced stressors, similar to its superior protection against ROS-associated chilling stress of the sensitive boar spermatozoa (Waberski et al., 2019; O'Brien et al., 2021). Stressors associated with the PDI approach may result from single or combined spermicidal effects of the PS, illumination, and the PDI-induced ROS production.

It is suggested that typical semen extender components, such as EDTA, make cell walls more permeable, thereby supporting the phototoxicity of the PS (Pohl et al., 2016). Different extender compositions may explain variation in PDI efficiency between extenders. Recipes for more recently marketed commercial extenders are no longer published, so the mechanism of PDI-extender interaction remains unknown.

The dark controls used here confirm previous studies showing that the APrem extender has intrinsic antimicrobial activity. The PDI enlarges this effect, which is especially apparent by inactivating *Proteus vulgaris*, a multi-drug resistant bacterium that grows during the long-term storage of boar semen at 17°C (Delgado-Bermúdez et al., 2020; Luther et al., 2023). It should be noted that the effective PS concentration and illumination intensity were relatively low compared to the PDI application in aqueous systems. In different water matrixes consisting of freshwater and industrial water, an inactivation of *E.coli* below the detection limit was only achieved with up to 60 min of light treatment with 50 mW/cm^2^ and a porphyrine concentration of 5 μM (Bartolomeu et al., 2023), whereas dose–response studies of *E. coli* in PBS indicate that 2 μM TMPyP and an illumination with 10.8 W/cm^2^ for 10 min reduces CFU of *E. coli* by 6 log_10_ steps (Muehler et al., 2022). When using PDI on semen, the illumination stress must be kept as low as possible to protect the spermatozoa. Here, we showed that illumination for only 90 s in the presence of 2 μM TMPyP was sufficient to inactivate bacteria during long-term semen storage. The proof-of-principle testing *in vivo* demonstrated that semen undergoing photodynamic treatment maintained its fertilizing capacity with a high number of offspring in two sows. The third inseminated sow also presumably became pregnant, which, for unknown reasons, was not carried to term, a phenomenon which is well known within the first 30 days of gestation in sows (Koketsu et al., 2017). At this early experimental stage of the innovative PDI use for semen decontamination, further adaptations of the technique for application in semen doses with higher volumes (typically 60–100 mL) are required before field insemination trials can be conducted. Ideally, this would be achieved by all around evenly illuminating extended semen doses in transparent bags or tubes.

In conclusion, the PDI has been established as an innovative method to prevent bacterial growth in extended semen. The photodynamic treatment was successfully adapted to the specific extender environment and for long-term semen storage at 17°C. The results achieved a balance between the inactivation of bacteria and maintenance of sperm fertility. Hence, a potential alternative to the use of antibiotics, which can cause resistance in semen extenders, has been found. However, further testing and adaptation are needed for practical application. Given the low cost of the PS used here and the affordability of LED light sources, the prospect of implementing the novel technology in AI centers is realistic.

## Data availability statement

The original contributions presented in the study are included in the article/Supplementary material, further inquiries can be directed to the corresponding authors.

## Ethics statement

The animal study was approved by European Commission Directive for Pig Welfare following the ARRIVE guidelines. The study was conducted in accordance with the local legislation and institutional requirements.

## Author contributions

A-ML: Conceptualization, Data curation, Formal analysis, Investigation, Methodology, Visualization, Writing – original draft, Writing – review & editing. MV: Formal analysis, Investigation, Visualization, Writing – review & editing. CB: Formal analysis, Investigation, Visualization, Writing – review & editing. LF: Investigation, Visualization, Writing – review & editing. IM: Data curation, Formal analysis, Investigation, Visualization, Writing – review & editing. HO: Formal analysis, Investigation, Visualization, Writing – review & editing. SH: Conceptualization, Funding acquisition, Methodology, Project administration, Resources, Supervision, Visualization, Writing – original draft, Writing – review & editing. DW: Conceptualization, Funding acquisition, Methodology, Project administration, Resources, Supervision, Writing – original draft, Writing – review & editing.
