# Supplementary figures and images for: Fertility after photodynamic inactivation of bacteria in extended boar semen
Source: Front Microbiol. 2024 Aug 7;15:1429749. doi: 10.3389/fmicb.2024.1429749 (PMC11335528; doi:10.3389/fmicb.2024.1429749)

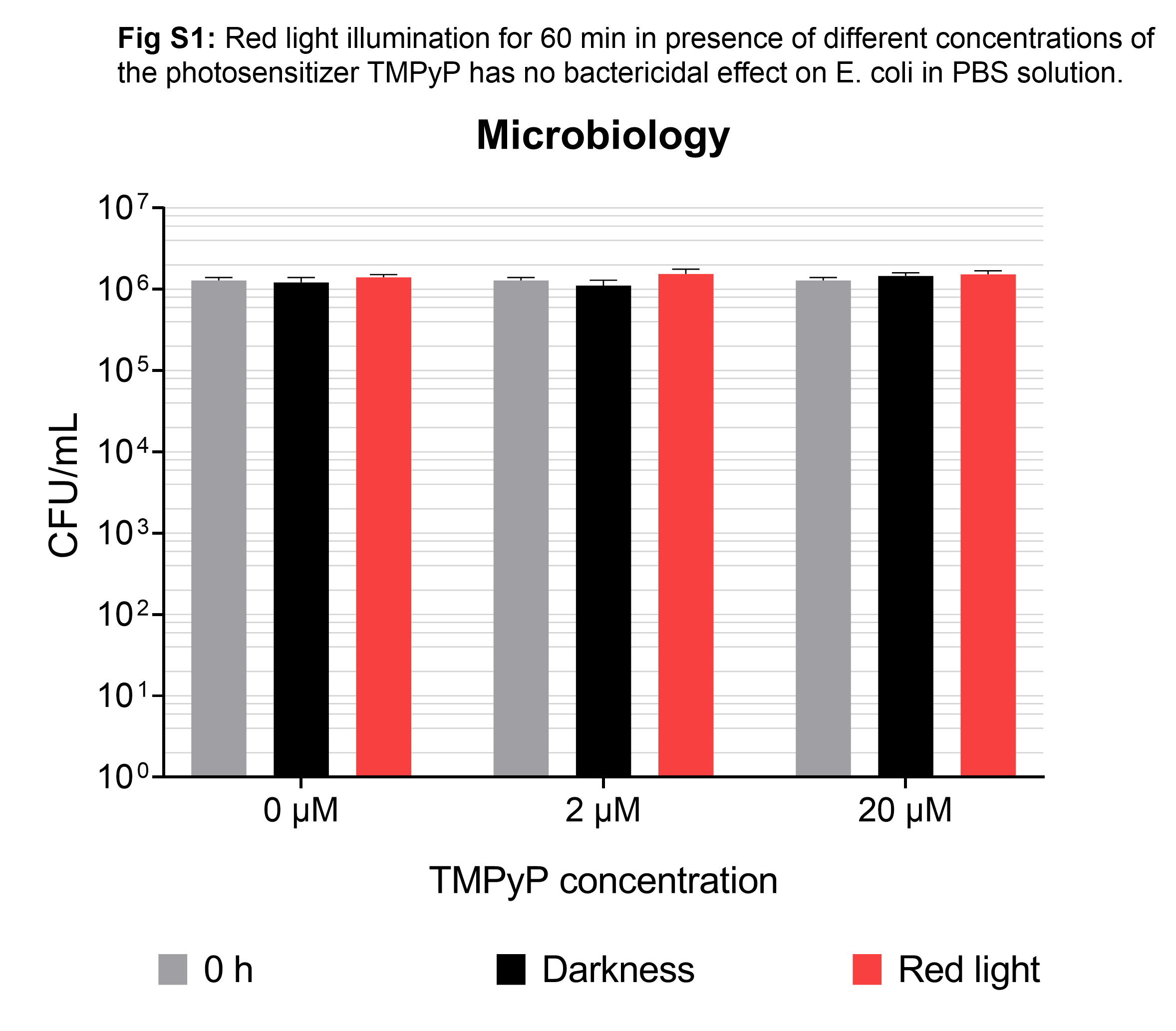

Supplement: Supplementary file 1 [file Image_1.tif]

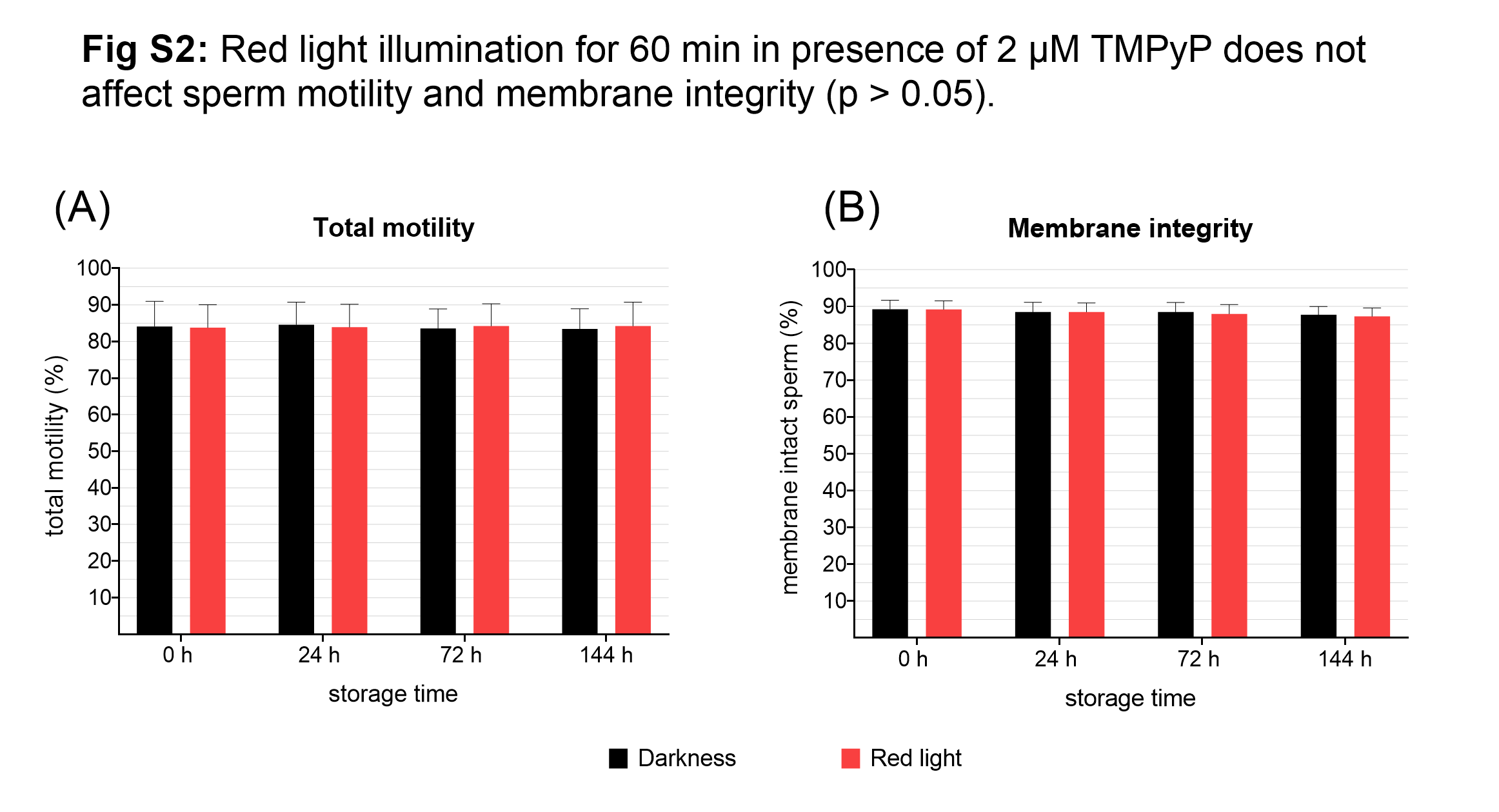

Supplement: Supplementary file 2 [file Image_2.tif]

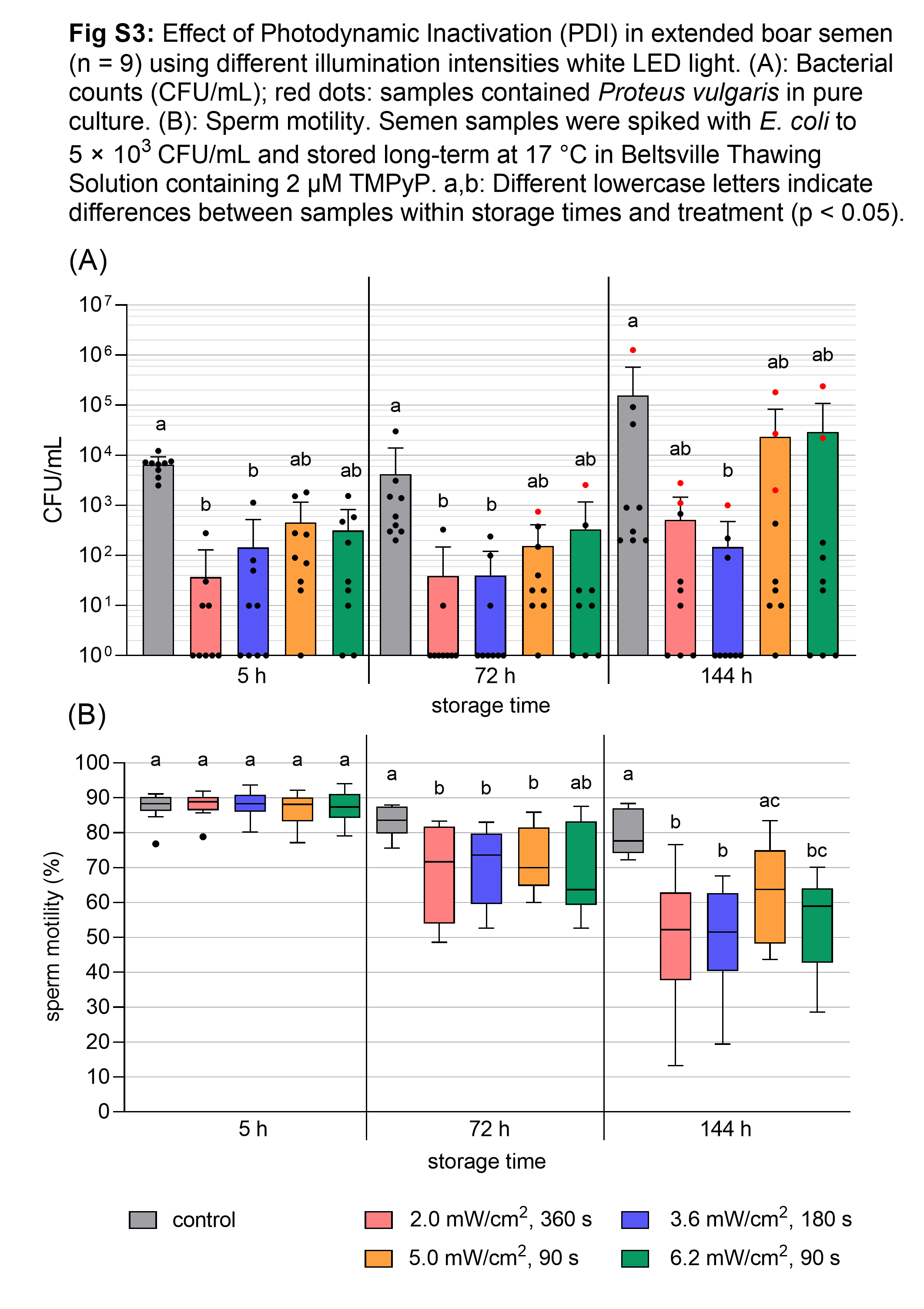

Supplement: Supplementary file 3 [file Image_3.tif]
