# Supplementary material for: Fertility after photodynamic inactivation of bacteria in extended boar semen
Source: Front Microbiol. 2024 Aug 7;15:1429749. doi: 10.3389/fmicb.2024.1429749 (PMC11335528; doi:10.3389/fmicb.2024.1429749)
Supplement: Supplementary file 4 [file Table_1.DOCX]

**Table S1.** Effect of Photodynamic Inactivation (PDI) in extended boar semen using different illumination intensities white LED light. Sperm kinematics were evaluated with computer-assisted semen analysis (AndroVision®, Version 1.2, Minitüb GmbH) during long-term storage at 17 °C in Beltsville Thawing Solution (BTS) containing 2 µM TMPyP. The extended semen was spiked with 5 × 10^3^ CFU/mL *E. coli*. a-c: Different lowercase letters indicate differences between samples within storage times and treatment (*p* < 0.05). A-C: Different uppercase letters indicate differences between storage time points within samples (*p* < 0.05). Data are means and SEM (n=9).

| **Progressive motility (%)** | control | 80.9 ± 2.3^a,A^ | 74.5 ± 3.2^a,A^ | 67.0 ± 4.9^a,A^ |
| --- | --- | --- | --- | --- |
|  | 2 mW/cm^2^, 360 s | 80.7 ± 2.1^a,A^ | 57.9 ± 4.4^b,B^ | 37.0 ± 5.7^b,C^ |
|  | 3.6 mW/cm^2^, 180 s | 81.2 ± 1.7^a,A^ | 60.2 ± 4.0^b,B^ | 37.6 ± 4.6^b,C^ |
|  | 5 mW/cm^2^, 90 s | 80.2 ± 2.3^a,A^ | 63.6 ± 2.8^b,B^ | 50.7 ± 5.1^ac,B^ |
|  | 6.2 mW/cm^2^, 90 s | 81.7 ± 1.9^a,A^ | 61.7 ± 4.7^b,B^ | 43.0 ± 4.6^bc,C^ |
| **Velocity curvilinear line (µm/s)** | control | 156.7 ± 14.5^a,A^ | 136.0 ± 5.5^a,A^ | 138.2 ± 10.8^a,A^ |
|  | 2 mW/cm^2^, 360 s | 143.5 ± 18.3^ab,A^ | 136.8 ± 7.8^a,A^ | 129.8 ±11.2^a,A^ |
|  | 3.6 mW/cm^2^, 180 s | 145.1 ± 17.3^ab,A^ | 145.1 ± 8.2^a,A^ | 134.8 ± 11.8^a,A^ |
|  | 5 mW/cm^2^, 90 s | 150.2 ± 18.2^ab,A^ | 136.0 ±6.7^a,A^ | 143.5 ± 11.9^a,A^ |
|  | 6.2 mW/cm^2^, 90 s | 140.9 ± 14.7^b,A^ | 144.7 ± 5.0^a,A^ | 140.4 ± 9.3^a,A^ |
| **Amplitude of lateral head displacement**  **(µm)** | control | 1.36 ± 0.16^a,A^ | 1.13 ± 0.04^a,A^ | 1.16 ± 0.08^a,A^ |
|  | 2 mW/cm^2^, 360 s | 1.27 ± 0.20^ab,A^ | 1.20 ± 0.06^a,A^ | 1.28 ± 0.10^ab,A^ |
|  | 3.6 mW/cm^2^, 180 s | 1.24 ± 0.18^ab,A^ | 1.26 ± 0.07^a,A^ | 1.29 ± 0.11^b,A^ |
|  | 5 mW/cm^2^, 90 s | 1.26 ± 0.18^ab,A^ | 1.19 ± 0.05^a,A^ | 1.30 ± 0.10^b,A^ |
|  | 6.2 mW/cm^2^, 90 s | 1.16 ± 0.14^b,A^ | 1.24 ± 0.04^a,A^ | 1.29 ± 0.08^b,A^ |
| **Beat Cross Frequency (Hz)** | control | 31.7 ± 1.4^a,AB^ | 32.1 ± 1.0^a,A^ | 30.3 ± 1.1^a,AB^ |
|  | 2 mW/cm^2^, 360 s | 31.3 ± 1.6^a,A^ | 29.0 ± 1.0^b,A^ | 24.4 ± 0.8^b,B^ |
|  | 3.6 mW/cm^2^, 180 s | 31.8 ± 1.6^a,A^ | 29.7 ± 0.9^ab,A^ | 25.2 ± 0.8^b,B^ |
|  | 5 mW/cm^2^, 90 s | 32.4 ± 1.6^a,A^ | 29.2 ± 0.8^b,A^ | 27.3 ± 0.9^bc,B^ |
|  | 6.2 mW/cm^2^, 90 s | 32.9 ± 1.5^a,A^ | 29.9 ± 0.8^ab,A^ | 27.4 ± 1.0^ac,B^ |
